# Counterfactual Reasoning in Non-psychotic First-Degree Relatives of People with Schizophrenia

**DOI:** 10.3389/fpsyg.2016.00665

**Published:** 2016-05-09

**Authors:** Auria Albacete, Fernando Contreras, Clara Bosque, Ester Gilabert, Ángela Albiach, José M. Menchón, Benedicto Crespo-Facorro, Rosa Ayesa-Arriola

**Affiliations:** ^1^Psychiatry Department, Bellvitge University Hospital – Institut d’Investigació Biomèdica de BellvitgeBarcelona, Spain; ^2^Department of Clinical Sciences, School of Medicine, University of BarcelonaBarcelona, Spain; ^3^Carlos III Health Institute, Centro de Investigación Biomédica en Red de Salud MentalBarcelona, Spain; ^4^Fundació per a la Investigació i Docència María Angustias Giménez, Germanes HospitalàriesBarcelona, Spain; ^5^Mental Health Unit L’Hospitalet, SAP Delta Llobregat – Catalan Institute of HealthBarcelona, Spain; ^6^Psychiatry Department, Marqués de Valdecilla University Hospital – Instituto de Investigación Marqués de ValdecillaSantander, Spain

**Keywords:** schizophrenia, counterfactual thinking, reasoning, endophenotype, first-degree relatives

## Abstract

Counterfactual thinking (CFT) is a type of conditional reasoning that enables the generation of mental simulations of alternatives to past factual events. Previous research has found this cognitive feature to be disrupted in schizophrenia (Hooker et al., 2000; Contreras et al., 2016). At the same time, the study of cognitive deficits in unaffected relatives of people with schizophrenia has significantly increased, supporting its potential endophenotypic role in this disorder. Using an exploratory approach, the current study examined CFT for the first time in a sample of non-psychotic first-degree relatives of schizophrenia patients (*N* = 43), in comparison with schizophrenia patients (*N* = 54) and healthy controls (*N* = 44). A series of tests that assessed the “causal order effect” in CFT and the ability to generate counterfactual thoughts and counterfactually derive inferences using the Counterfactual Inference Test was completed. Associations with variables of basic and social cognition, levels of schizotypy and psychotic-like experiences in addition to clinical and socio-demographic characteristics were also explored. Findings showed that first-degree relatives generated a lower number of counterfactual thoughts than controls, and were more adept at counterfactually deriving inferences, specifically in the scenarios related to regret and to judgments of avoidance in an unusual situation. No other significant results were found. These preliminary findings suggest that non-psychotic first-degree relatives of schizophrenia patients show a subtle disruption of global counterfactual thinking compared with what is normally expected in the general population. Due to the potential impact of such deficits, new treatments targeting CFT improvement might be considered in future management strategies.

## Introduction

Counterfactual Thinking (CFT) is a specific type of conditional reasoning related to mental simulations of past events generally triggered to a great extent by negative outcomes. In other words, and to quote Van Hoeck et al. (2015, p. 1), CFT refers to the “remarkable ability to infer how an event might have unfolded differently, without directly experiencing this alternative reality." In this way, most people compare the actual result of the event with “what might have been" by generating different hypothetical outcomes “if only" an alternative event had taken place (Byrne, 2016). For instance, in the fictional scenario where John has failed an important test, he could automatically generate a counterfactual thought like, *If I had studied more, I could have passed the test.*

Concerning CFT’s neuroanatomical correlates, fMRI studies suggest that prefrontal cortex (PFC) regions are the primary regions activated in people engaged in counterfactual reasoning tasks, although other regions have been found to be related such as the temporal lobes, the left temporal gyrus, and the left cerebellum (Barbey et al., 2009; Van Hoeck et al., 2012). Counterfactual reasoning supports adaptive behavior by enabling us to learn from past experiences (Epstude and Roese, 2008) and by modulating emotional state (Roese and Olson, 1997), promoting creativity (Markman et al., 2007), and supporting future planning and prediction (Smallman and Roese, 2009), in addition to playing a behavior-regulating function that influences behavioral changes and performance improvement (Epstude and Roese, 2008). Furthermore, CFT seems to be related to specific cognitive biases such as the hindsight bias—enhancing memory distortions that contribute to suboptimal decision-making (Roese and Olson, 1996) and to Theory of Mind (ToM) deficits involved in the development of false belief (Byrne, 2016).

Studies of subtle cognitive alterations in unaffected relatives of schizophrenia patients have significantly increased over the last decade in an effort to confirm the hypothesis that these deficiencies might be potential endophenotypes for this disorder (Gottesman and Gould, 2003; Sitskoorn et al., 2004). Contributing to the etiology of schizophrenia (Cardno et al., 1999; Cannon et al., 2000; Byrne et al., 2003), these deficits seem to meet the fifth criterion for endophenotype validation in psychiatry since they are found to a lesser degree in the unaffected relatives of people with this disorder (Gottesman and Gould, 2003). Such deficiencies include alterations in declarative and working memory, sustained attention, verbal fluency, perceptual-motor speed, and certain executive functions (Sitskoorn et al., 2004; Szöke et al., 2005; Snitz et al., 2006). In addition, cognitive biases related to particular symptoms of schizophrenia, such as the data gathering bias known as “jumping to conclusions,” have also been described among non-psychotic relatives of patients with schizophrenia (Van Dael et al., 2006; Broome et al., 2007). Regarding social cognition, the data remain inconsistent (Green et al., 2008). Some studies have found evidence of alterations in these probands compared with normal controls (Janssen et al., 2003; Bediou et al., 2007; Lavoie et al., 2013; Cella et al., 2015), whereas other studies have shown no differences at all (Kelemen et al., 2004; Loughland, 2004). Similar deficits have also been observed both in unaffected first-degree relatives high in schizotypy (Chen et al., 1998; Laurent et al., 2000; Vollema and Postma, 2002) and in healthy individuals that have reported psychotic-like experiences (PLEs; Kelleher and Cannon, 2011).

Currently, there is general agreement that neurocognition is a key feature of schizophrenia with deficits in all cognitive domains (Heinrichs and Zakzanis, 1998). Such deficits have already been recorded in the early stages of the disorder (Censits et al., 1997; Keefe et al., 2006; Crespo-Facorro et al., 2011). Thus, given that schizophrenia seems to be related, at least in part, to a PFC dysfunction (Goldman-Rakic, 2011; Jackowski et al., 2012), and that this neurocognitive impairment seems to be the single strongest correlate of these patients’ real-world functioning (Green, 1996; Fett et al., 2011), it is not surprising that studies exploring counterfactual reasoning in these patients have emerged over recent years (Hooker et al., 2000; Contreras et al., 2016). Accordingly, the results of such research have revealed disruption in these patients’ abilities to generate counterfactual thoughts, to attribute causality through CFT, as well as to counterfactually derive inferences in the face of different fictional social scenarios. The further study of these CFT disruptions in the schizophrenia spectrum has been encouraged since it might help in the understanding of these patients’ day-to-day challenges, or eventually provide a new diagnostic tool or even a new target for treatment (Van Hoeck et al., 2015). In keeping with this goal, one possible approach might be through the identification of these cognitive endophenotypes among individuals who have no clinical needs but are at risk for psychosis, such as unaffected first-degree relatives of people with schizophrenia. Research on this topic has become an important area of investigation in recent years not only for providing critical information about the pathophysiology of the disorder but also for its potential to direct early interventions and prevention programs among both schizophrenia patients and these at-risk individuals.

Using an exploratory approach, the current study reports the assessment of CFT in a sample of non-psychotic first-degree relatives of people with schizophrenia. To our knowledge, this is the first time that counterfactual reasoning has been explored among this group and compared to schizophrenia patients and healthy controls. CFT was quantitatively evaluated using different methods of assessment including (1) the generation of counterfactual thoughts, (2) the “causal order effect” on CFT (Wells et al., 1987), and (3) the ability to make counterfactual-derived inferences, assessed using the Counterfactual Inference Test (CIT; Hooker et al., 2000). Potential associations with measures of neurocognition and social cognition, level of schizotypy and PLEs, as well as with any particular socio-demographic characteristic, were further assessed.

## Materials and Methods

### Participants

A total of 141 participants—54 patients with schizophrenia, 43 non-psychotic first-degree relatives, and 44 healthy controls—all fluent in Spanish and between 19 and 66 years of age, were included in the study after an initial inclusion interview in which an informed consent form was signed and mental and personality disorders were assessed using the structured clinical interview for DSM-IV Axis I Disorders (SCID-I; First et al., 1997) and Axis II Personality Disorders (SCID-II; First et al., 1994). The sample was recruited from the outpatient services of the Psychiatry Department of Bellvitge University Hospital, the Polyvalent Mental Health Unit (Benito Menni CASM), and the Mental Health Unit of L’Hospitalet de Llobregat (Catalan Institute of Health). Potential participants were excluded if they had a history of head trauma involving loss of consciousness, an organic disease with mental repercussions, or an estimated Intelligence Quotient (IQ) below 70. All study procedures were approved by the Clinical Research Ethics Committee of the Ciutat Sanitària de Bellvitge (CEIC Bellvitge).

First-degree relatives—19 parents, 19 siblings, and 5 offspring—of schizophrenia patients of the three collaborating units were also sampled. Family members were excluded if they had a history of a psychotic disorder or substance abuse. All schizophrenia patients met DSM-IV-TR criteria (American Psychiatric Association, 2000), were in remission as defined by Andreasen et al. (2005), and had not undergone electroconvulsive therapy in the last 6 months. Participants with other Axis I disorders were excluded. Healthy control participants were recruited from hospital employees; exclusion criteria were a previous history of personal (Axis I and Axis II) or family psychiatric illness or substance use disorder.

### Measures and Procedures

#### Socio-demographic and Clinical Measures

Socio-demographic data were collected for all participants, including gender, age, years of education, current occupation, and civil status. Laterality was assessed by means of the Edinburgh Handedness Inventory (Oldfield, 1971). Estimated IQ was calculated using a combined score from the Vocabulary and Block Design subtests from the Wechsler Adult Intelligence Scale battery III (Sattler, 2001; Wechsler, 2001).

Symptoms and severity of illness were assessed using the Positive and Negative Syndrome Scale (PANSS; Kay et al., 1987; Peralta and Cuesta, 1994), the Montgomery–Asberg Depression Rating Scale (MADRS; Montgomery and Asberg, 1979; Lobo et al., 2002), the Clinical Global Impression-Severity Scale (CGI-S; Guy, 1976), and the Scale to Assess Unawareness of Mental Disorder (SUMD; Amador and Strauss, 1990; Ruiz et al., 2008). Level of functioning was assessed with the Global Assessment of Functioning Scale (GAF; American Psychiatric Association, 2000). Pharmacological treatment was recorded, and antipsychotic daily dose equivalents of chlorpromazine were calculated (Kane et al., 2003).

#### Counterfactual Thinking Evaluation

Counterfactual thinking was examined using a set of three different measures in the following order: (1) the “causal order effect,” (2) the generation of counterfactual thoughts, and (3) the ability to counterfactually derive inferences.

To begin with, two experiments framed on the research paradigm originally designed by Wells et al. (1987) were carried out to examine the first two measures. For further information about this procedure, the reader is referred to the work of these aforementioned authors, but, in brief, the procedure consisted of reading aloud to the probands a fictional scenario of four consecutive independent events that resulted in a negative outcome. In order to avoid the first event bias, the researcher randomly changed the order of the events using a 4 × 4 Latin square design. Thus, the scenario provided the frame for the two experiments described below.

##### Experiment 1: The causal order effect

In this experiment, participants were asked to choose which one of the four events was the most probable cause of the negative outcome of the scenario—in other words, the event they would select in order to undo the final result. This procedure was based on previous research that has described how the general population usually chooses the first of a chain of events as the main determinant event, even though these events are equal and objectively none is more crucial than the others for the final negative outcome (Segura et al., 2002). Thus, this effect explains how the focus of CFT tends to be influenced by the order in which the information is presented. To complete this experiment, participants had to choose a specific event from the sequence. Those who, even when encouraged, were still unable to choose one of the events, were directly assigned the response type “reasoning blocking.” This was done to ensure that these responses were not considered as missing data. The time given to participants to complete this experiment was 60 s. Researchers recorded each participant’s answer.

##### Experiment 2: Generation of counterfactual thoughts

The ability to spontaneously generate counterfactual thoughts for the purpose of avoiding the final negative outcome was assessed by asking the participants to say aloud as many alternatives as possible. These counterfactual thoughts could be original alternatives (e.g., “If only I had called and made a reservation in advance”) or alternatives that changed one of the “unfortunate” events (e.g., “If only I hadn’t been speeding”). All discrete responses given were recorded by two independent researchers, who filtered which answers were real counterfactual thoughts and which ones were illogical or bizarre (e.g., “I continued sleeping”).

##### Counterfactual Inference Test (CIT)

Originally developed by Hooker et al. (2000), the CIT was administered to measure ability to generate counterfactually derived inferences. This is a multiple-choice, self-reporting instrument designed to evaluate the influence of different specific characteristics of a situation when individuals generate counterfactual inferences. The CIT is based on previous research that described how CFT is enhanced when encountering events with outcomes preceded by unusual rather than typical actions (Kahneman and Tversky, 1982), or events that seem “almost” to have occurred—either spatially or temporally (Kahneman and Varey, 1990). CFT can also influence an individual’s affective and judgmental reaction to these situations by enhancing or diminishing these reactions (Kahneman and Tversky, 1982; Kahneman and Varey, 1990).

Thus, the CIT presents four scenarios in which two events with similar outcomes are experienced by two different subjects. However, the circumstances between the events differ such that one of the subjects should think “if only” to a greater extent than the other. For each situation, three possible answers are presented: a target counterfactual response (the option where CFT is activated to a greater extent), a non-target response (the option where CFT is also activated but less intensely), and a “same/can’t tell” answer if the participant considers none of the previous options to be suitable (the option where CFT is not activated at all; see **Table 1**). Each scenario in the test is given a maximum score of 1 if the subject chooses the target counterfactual response; if the subject chooses any of the other answers, the score given is zero. The total score, therefore, may range between 0 and 4, with greater values indicating a counterfactual response closer to a normative pattern.

**Table 1:** The Counterfactual Inference Test (Hooker et al., 2000).

Scenario	Response
(1) Reaction of upset (affective) in response to a spatial “nearly happened” event *Janet is attacked by a mugger only 10 m from her house. Susan is attacked by a mugger 1 km from her house. Who is more upset by the mugging?*	**(a) Janet** (b) Susan (c) Same/Can’t tell
(2) Reaction of regret (affective) in response to an “unusual” event *Anna gets sick after eating at a restaurant she often visits. Sarah gets sick after eating at a restaurant she has never visited before. Who regrets their choice of restaurant more?*	(a) Anna **(b) Sarah** (c) Same/Can’t tell
(3) Reaction of rumination (judgmental) in response to a temporal “nearly happened” event *Jack misses his train by 5 min. Ed misses his train by more than an hour. Who spends more time thinking about the missed train?*	(a) Ed **(b) Jack** (c) Same/Can’t tell
(4) Reaction of avoidance (judgmental) in response to an “unusual” event *John gets into a car accident while driving on his usual way home. Bob gets into a car accident while trying a new way home. Who thinks more about how his accident could have been avoided?*	**(a) Bob** (b) John (c) Same/Can’t tell

*Typical pattern of responses—that is, the target counterfactual responses—are indicated in boldface (Hooker et al., 2000).*

#### Neuropsychological Evaluation

Cognitive function was assessed using a comprehensive battery of 13 standardized neuropsychological tests, designed to encompass all cognitive dimensions proposed in the MATRICS battery (Green et al., 2004). The tests are summarized in **Table 2**.

**Table 2:** Neuropsychological Test Battery.

Cognitive domain	Test
Laterality	Edinburgh Handedness Inventory (Oldfield, 1971)
Estimated IQ	Wechsler Adult Intelligence Scale-III, Vocabulary Test (Wechsler, 2001)
	Wechsler Adult Intelligence Scale-III, Block Design Test (Wechsler, 2001)
Attention	Continuous Performance Test-II; CPT (Conners, 2000)
Processing speed	Trail Making Test – Form A (Reitan, 1958)
	Wechsler Adult Intelligence Scale-III, Symbol Coding Test (Wechsler, 2001)
	Stroop Test, word-color (Golden, 1978)
Executive function	Trail Making Test – Form B (Reitan, 1958)
	Stroop Test, word-color interference effect (Golden, 1978)
	Controlled Oral Word Association Test, FAS-Test (Loonstra et al., 2001)
	Test Barcelona, Animal Words (Peña-Casanova, 1990)
	Wisconsin Card Sorting Test, WCST-128 (Heaton et al., 1993)
	Tower of London Test (Culbertson and Zillmer, 2001)
Working memory	Wechsler Adult Intelligence Scale-III, Digit Span Test (Wechsler, 2001)
	Wechsler Adult Intelligence Scale-III, Letter-Number Sequencing Test (Wechsler, 2001)
Verbal memory	California Verbal Learning Test, Spanish version –TAVEC (Benedet and Alejandre, 1998)
Visual memory	Wechsler Memory Scale-III, Visual reproduction Tests I and II (Wechsler, 1997)
Social cognition	Mayer–Salovey–Caruso Emotional Intelligence Test, MSCEIT (Extremera and Fernández-Berrocal, 2009)
	Internal, Personal, and Situational Attributions Questionnaire, IPSAQ (Kinderman and Bentall, 1996)

#### Evaluation of Schizotypy and Psychotic-like Experiences

Levels of schizotypy and PLEs were assessed among relatives and controls using the Schizotypal Personality Questionnaire-Brief (SPQ-B; Raine and Benishay, 1995; Spanish adaptation by Mata et al., 2005) and the Community Assessment of Psychotic Experiences-42 (CAPE-42; Stefanis et al., 2002; Spanish adaptation by Fonseca-Pedrero et al., 2012), respectively.

### Statistical Analysis

For the descriptive analyses, absolute and relative frequencies were calculated for categorical variables. Continuous variables were assessed using the mean (M) and standard deviation (SD) for normally distributed variables, and the median and range for non-normally distributed variables. To detect differences between groups, Fisher’s exact test and χ^2^ were used for categorical data, whereas group means were compared using one-way analysis of variance (ANOVA) followed by a Tukey test for *post hoc* analyses. The Kruskal–Wallis test was used for non-normally distributed data. Multivariate linear regression analyses were done to assess significant differences between groups for all CFT measures, adjusted for age, gender, and estimated IQ, as well as to explore potential associations between these measures and variables of neurocognition and social cognition, schizotypy, PLEs, and socio-demographic and clinical characteristics. A value of *p* < 0.05 was considered statistically significant. All analyses were conducted using R 3.1.3.

## Results

### Socio-demographic and Clinical Characteristics

Socio-demographic characteristics are summarized in **Table 3**. The results of the analyses revealed the group of relatives to be older than the rest of the sample, and the group of patients to have a higher proportion of single and retired individuals, and a lower proportion of women and a lower estimated IQ. Regarding clinical characteristics, the patients exhibited mild levels of symptom severity on the PANSS total score (*M* = 65.26, *SD* = 7.80), on the MADRS (*M* = 11.65, *SD* = 6.24), on the SUMD (*M* = 5.20, *SD* = 2.68), and on the CGI-SCH (*M* = 3.30, *SD* = 0.50). The median GAF score was 70 (range = 50–80); the average length of illness was 16.32 years (*SD* = 10.42); and the mean daily dose of antipsychotic treatment taken was 650.94 mg/day (*SD* = 468.75; chlorpromazine equivalents).

**Table 3:** Socio-demographic characteristics of the sample and comparison between groups.

	Schizophrenia patients (*n* = 54)	First-degree relatives (*n* = 43)	Healthy controls (*n* = 44)	*p*-value
Gender, male: *n* (%)	37 (68.5)	19 (44.2)	21 (47.7)	0.031
Age, years	41.4 (11.1)	50.7 (12.2)	45.6 (12.6)	0.002
Educational level, years	9.7 (2.3)	9.9 (3.5)	10.3 (2.7)	0.502
Employment status: *n* (%)				<0.0001
Employed/Student	5 (9.3)	25 (58.1)	31 (70.5)	
Unemployed	12 (22.2)	12 (27.9)	11 (25.0)	
Retired	37 (68.5)	6 (14.0)	2 (4.5)	
Civil status: *n* (%)				0.000
Single	39 (72.2)	11 (25.6)	13 (29.5)	
Married	10 (18.5)	28 (65.1)	21 (47.7)	
Divorced	5 (9.3)	4 (9.3)	7 (15.9)	
Widowed	0 (0.0)	0 (0.0)	3 (6.8)	
Handedness, right: (%)	87.0	90.7	90.9	0.378
Estimated IQ	94.70 (11.57)	104.56 (11.51)	105.36 (14.50)	0.000

*Values presented as means (standard deviation) unless otherwise specified.*

### Counterfactual Thinking Evaluation

#### Experiment 1: The Causal Order Effect

No statistically significant differences were found for this experiment in the general pattern of responses between the first-degree relatives, the schizophrenia patients and the healthy subjects (χ^2^ = 3.19, *p* = 0.922; **Table 4**). In addition, the proportion of participants unable to choose any of the four events (that is, the “reasoning blocking” response) was similar between groups (χ^2^ = 0.40, *p* = 0.820). Nonetheless, the results showed a tendency among the healthy controls to choose the first event more frequently than the other groups (29.5% versus 27.9% of the relatives and 24.1% of the patients).

**Table 4:** The causal order effect (Experiment 1): Descriptive and comparative analysis between groups.

	Schizophrenia patients (*n* = 54)	First-degree relatives (*n* = 43)	Healthy controls (*n* = 44)	χ^2^-test (*p*-value)
**Experiment 1: The causal order effect**				
Order of the events, *n* (%)				3.19 (0.922)
1st	13 (24.1)	12 (27.9)	13 (29.5)	
2nd	9 (16.7)	7 (16.3)	11 (25.0)	
3rd	10 (18.5)	5 (11.6)	6 (13.6)	
4th	14 (25.9)	12 (27.9)	9 (20.5)	
Reasoning blocking^a^	8 (14.8)	7 (16.3)	5 (11.4)	
1st vs. 2nd, 3rd, 4th, reasoning blocking	24.1/75.9	27.9/72.1	29.5/70.5	0.40 (0.820)

*^a^Unable to choose any event.*

#### Experiment 2: Generation of Counterfactual Thoughts

**FIGURE 1:**
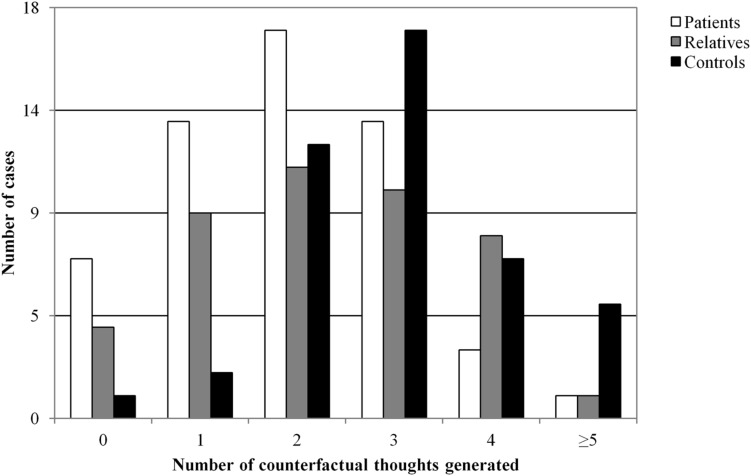
**Number of counterfactual thoughts generated by groups in the study (Experiment 2)**.

**Figure 1** presents the results of this experiment. *Post hoc* analysis revealed that first-degree relatives generated a significantly lower number of counterfactual thoughts than the healthy control group (*p* = 0.030). This difference was also observed between the healthy controls and the schizophrenia patients (*p* = 0.0001). However, when adjusted for age, gender, and estimated IQ, these differences maintained only a borderline level of statistical difference (*F* = −0.51, *p* = 0.061). Nevertheless, and as expected, patients generated fewer counterfactual thoughts compared with the other groups (χ^2^ = 16.15, *p* = < 0.001). Differences between controls and patients remained significant even when adjusted (*F* = –0.79, *p* = 0.004).

#### Counterfactual Inference Test

Analyses of group differences on the CIT total score revealed statistically significant differences in favor of the relatives compared with the controls when adjusted for age, gender, and estimated IQ (*F* = 0.73, *p* = 0.005), but not for the patients compared to the controls (*F* = 0.19, *p* = 0.446; **Table 5**). When examining each particular scenario of the test separately, the results revealed that a significantly higher proportion of first-degree relatives chose the target counterfactual response than in the other groups for the specific situations related to regret in the face of an unusual event (*p* = 0.047; Scenario 2) and related to judgments of avoidance also in response to an unusual event (*p* = 0.036; Scenario 4). This difference was also observed in Scenario 1 (*p* = 0.013), but this time in favor of the schizophrenia patients. No significant differences were found regarding Scenario 3 (**Table 6**).

**Table 5:** Descriptive and comparative analysis between groups on the CIT total score.

	Schizophrenia patients (*n* = 54)	First-degree relatives (*n* = 43)	Healthy controls (*n* = 44)	Kruskal–Wallis test (*p*-value)
Total score, *n* (%)				χ^2^ = 5.28 (0.071)
0	7 (13)	4 (9)	6 (14)	
1	12 (23)	9 (21)	13 (30)	
2	19 (36)	9 (21)	15 (34)	
3	10 (19)	13 (30)	8 (18)	
4	5 (9)	8 (19)	2 (5)	

**Table 6:** Descriptive and comparative analysis between groups on the CIT scenarios.

	Schizophrenia patients (*n* = 54)	First-degree relatives (*n* = 43)	Healthy controls (*n* = 44)	Statistic	*p*-value
(1) Upset—spatial nearly happened event, *n* (%)				χ^2^ = 12.24^a^	0.014

Target counterfactual response	20 (37.7)	10 (23.3)	9 (20.5)		
Non-target response	9 (17.0)	5 (11.6)	1 (2.3)		
Same/can’t tell	24 (45.3)	28 (65.1)	34 (77.3)		

(2) Regret—unusual event, *n* (%)				χ^2^ = 9.60	0.048

Target counterfactual response	19 (35.8)	26 (60.5)	21 (47.7)		
Non-target response	20 (37.7)	7 (16.3)	8 (18.2)		
Same/can’t tell	14 (26.4)	10 (23.3)	15 (34.1)		

(3) Rumination—temporal nearly happened event, *n* (%)				χ^2^ = 2.91	0.573

Target counterfactual response	27 (50.9)	25 (58.1)	25 (56.8)		
Non-target response	18 (34.0)	10 (23.3)	9 (20.5)		
Same/can’t tell	8 (15.1)	8 (18.6)	10 (22.7)		

(4) Judgments of avoidance—unusual event, *n* (%)				χ^2^ = 12.24	0.036

Target counterfactual response	31 (58.5)	31 (72.1)	20 (45.5)		
Non-target response	11 (20.8)	2 (4.7)	7 (15.9)		
Same/can’t tell	11 (20.8)	10 (23.3)	17 (38.6)		

*Comparisons across groups in the study were made using the χ^2^-test unless otherwise specified. ^a^Fisher’s Exact Test.*

#### Associations with CFT Measures

The analyses showed no significant associations between CFT measures and any of the potential variables considered, including cognitive performance, schizotypy, and level of PLEs, in any of the three groups studied. Furthermore, among schizophrenia patients, clinical variables, including the daily dose of antipsychotic taken, were not related to CFT performance.

## Discussion

Counterfactual thinking is a specific type of conditional reasoning referring to the individual’s capacity to infer how an event might have displayed differently without directly experiencing this alternative scenario (Van Hoeck et al., 2015). Counterfactual thoughts are pervasive in everyday life and are involved in other processes such as problem-solving and learning from the experience (Epstude and Roese, 2008). The study of potential cognitive endophenotypes for schizophrenia has significantly increased in recent years in an effort to identify candidate genes associated with susceptibility for schizophrenia that could provide a more reliable index of liability than the illness itself (Gottesman and Shields, 1972; Cannon, 2005). Identifying these potential cognitive endophenotypes among at-risk samples would not only add knowledge about the pathophysiology of the disorder, but might also provide new guidelines for early interventions and prevention programs in schizophrenia patients and these at-risk individuals (Eack et al., 2010). With this purpose in mind, and based on previous research findings that demonstrated a global CFT impairment in schizophrenia (Hooker et al., 2000; Contreras et al., 2016), the present study assessed this type of reasoning for the first time in a sample of non-psychotic first-degree relatives, and compared them with a group of schizophrenia patients and healthy control subjects. Several striking results are discussed below in order of relevance.

Compared to what is normally expected in the general population, first-degree relatives were less skilful at generating spontaneous alternative representations using CFT in the face of a fictional situation with a negative outcome (Experiment 2). This alteration might be related to previous findings of a broad impairment in executive functions among relatives of schizophrenia patients, including difficulties in the ability to shift sets and to generate new alternatives of classification (Szöke et al., 2005). Moreover, in accordance with the fifth criterion for endophenotype validation, these preliminary results suggest deficits in the generation of counterfactual thoughts as a potential phenotypic marker of schizophrenia (Gottesman and Gould, 2003). Further research using a transdiagnostic approach may be warranted in order to properly examine the specificity of these deficits in schizophrenia and the genetic abnormalities underlying them.

When exploring a higher cognitive level of information processing, results on the CIT suggest that the first-degree relatives were in general more adept at deriving inferences from CFT when compared to the controls. In addition, when analyzing each item of the test in particular, the relatives were more proficient at making counterfactual-derived inferences in the specific scenarios assessing the effect of “unusualness” of the situation presented. This effect, which has previously been demonstrated in the general population, describes how an outcome preceded by an unusual rather than typical action influences CFT by enhancing it—as assessed in Scenarios 2 and 4 of the CIT (Kahneman and Tversky, 1982). Interestingly though, our findings suggest that the unusualness of the situation acted as a more intense CFT trigger for the relatives than for the other groups in the study. That is, the relatives tended to select the target-counterfactual response more frequently than the healthy subjects. Furthermore, a greater reaction of regret and judgments of avoidance was also observed among relatives compared with the controls. Again, this effect has also been observed in healthy controls (Kahneman et al., 1986), but in the present study, it seemed to be more pronounced among first-degree relatives. One possible explanation for both findings could rely on the presence of prominent schizotypy that could predispose these subjects to reacting more suspiciously than the controls. However, the present study explored this potential association using the SPQ-B and the CAPE-42, and no statistically significant results were found. This might be because the relatives that agreed to participate were probably the most compliant and most willing to take part in the research. This may have biased this group, since presumably relatives with prominent suspiciousness, significant interpersonal deficits, or subtle thought disorganization may have been less likely than healthier relatives to collaborate. This might also explain the lack of differences between relatives and controls on the SPQ-B and the CAPE-42 scores, along with the fact that none of the relatives met the criteria for personality disorder. Furthermore, results on the CIT might be conceptually linked to the study of cognitive biases that play a role in the development and maintenance of delusions in schizophrenia. Specifically, they might be related to the data gathering bias known as jumping to conclusions (e.g., inferring that if Sarah had not gone to a new restaurant she would not have got sick in Scenario 2), which has been observed not only among schizophrenia patients but also in their non-psychotic first-degrees relatives (Van Dael et al., 2006; Broome et al., 2007).

Concerning the causal order effect (Experiment 1), two results should be highlighted. Firstly, the general pattern of response when attributing causality under the effect order was similar between groups, and secondly, all three groups chose the first event in the sequence as the most decisive one. These results conflict with previous results on schizophrenia patients that revealed an alteration in the general pattern of response (Contreras et al., 2016). Framing their ideas in the *mental models theory*, Byrne et al. (2000) suggest that studying the aspects that people use to construct counterfactual alternatives is highly relevant, since these aspects, including causality, “give hints about the ‘joints’ of reality” in human beings (mental models). Thus, the present findings are more optimistic than previous results, since they show a preserved capacity among patients to attribute causality, which has to be beneficial for these patients’ functioning in daily life.

Finally, none of the cognitive functions assessed was related to any of the CFT measures recorded, despite the fact that the neuropsychological test battery was more extensive than in previous research. Consistent with previous findings in schizophrenia (Hooker et al., 2000; Contreras et al., 2016), the present results seem to support Van Hoeck et al.’s (2012) proposal of an integrated network of systems underlying CFT that might cut across different psychological domains. If there is not a distinctive counterfactual reasoning network, it seems logical that no specific neuropsychological test can detect this impairment. In fact, as other authors have already suggested, the present results reinforce the idea that this absence of significance might actually indicate that CFT could be related to more complex reasoning, social cognition and ToM-based abilities (Solca et al., 2015). Studies using other appropriate neuropsychological tests, as well as neuroimaging techniques, might be useful in solving this on-going debate.

The results of the present work need to be interpreted within the context of its limitations. First, as it was a pilot study, it involved a small number of subjects, which may have resulted in a lack of statistical power and greater chances of making a type II error, thus increasing the possibility that the study was not able to detect actual differences between groups. Second, there was potential bias in the representativeness of the first-degree relatives group—those willing to participate were probably the most “healthy” ones. In addition, there was no comparative analysis between types of relatives. However, because of the small sample size, this characteristic was not considered. Moreover, including parents and siblings all together may have made this group older on average than the controls and patients, and this fact might have had an impact on the reported differences. The authors tried to solve this issue by adjusting the results for age of the participants along with other possible confounding variables like gender and estimated IQ.

To our knowledge, this is the first study to report CFT performance in non-psychotic first-degree relatives of patients with schizophrenia. Compared with what is normally expected, relatives presented difficulties when spontaneously generating counterfactual alternatives to face a problem, and had a different pattern of reasoning when counterfactually deriving inferences. These findings represent a step forward in the investigation of counterfactual reasoning as a potential cognitive endophenotype for schizophrenia, and provide a new target for future early interventions and prevention programs not only for schizophrenia patients but also for these at-risk individuals. Further carefully designed family studies that incorporate other psychiatric populations and both molecular and neurobiological measures are still needed.

## Author Contributions

AA contributed to the management of the literature searches, design of the study, carried out the cognitive explorations, and undertook the statistical analysis. FC contributed in the management of the literature searches design of the study and the psychopathological evaluations. CB, EG, and ÁA contributed in the sample recruitment and psychopathological evaluations. RA-A contributed to the management of the literature searches and assisted with study design. JM supervised the data collection, contributed to the management of the literature searches, and assisted with study design. BC-F supervised the data collection, contributed to the management of the literature searches, and assisted with study design. All authors participated in the writing process, read and approved the final manuscript, and are in agreement to be accountable for all aspects of the work in ensuring that questions related to the accuracy or integrity of any part of the work are appropriately investigated and resolved.

## Conflict of Interest Statement

The authors declare that the research was conducted in the absence of any commercial or financial relationships that could be construed as a potential conflict of interest.
